# DAF-16 and Δ^9^ Desaturase Genes Promote Cold Tolerance in Long-Lived *Caenorhabditis elegans age-1* Mutants

**DOI:** 10.1371/journal.pone.0024550

**Published:** 2011-09-08

**Authors:** Fiona R. Savory, Steven M. Sait, Ian A. Hope

**Affiliations:** Institute of Integrative and Comparative Biology, Faculty of Biological Sciences, The University of Leeds, Leeds, United Kingdom; Semmelweis University, Hungary

## Abstract

In *Caenorhabditis elegans*, mutants of the conserved insulin/IGF-1 signalling (IIS) pathway are long-lived and stress resistant due to the altered expression of DAF-16 target genes such as those involved in cellular defence and metabolism. The three Δ^9^ desaturase genes, *fat-5*, *fat-6* and *fat-7*, are included amongst these DAF-16 targets, and it is well established that Δ^9^ desaturase enzymes play an important role in survival at low temperatures. However, no assessment of cold tolerance has previously been reported for IIS mutants. We demonstrate that long-lived *age-1(hx546)* mutants are remarkably resilient to low temperature stress relative to wild type worms, and that this is dependent upon *daf-16*. We also show that cold tolerance following direct transfer to low temperatures is increased in wild type worms during the facultative, *daf-16* dependent, dauer stage. Although the cold tolerant phenotype of *age-1(hx546)* mutants is predominantly due to the Δ^9^ desaturase genes, additional transcriptional targets of DAF-16 are also involved. Surprisingly, survival of wild type adults following a rapid temperature decline is not dependent upon functional *daf-16*, and cellular distributions of a DAF-16::GFP fusion protein indicate that DAF-16 is not activated during low temperature stress. This suggests that cold-induced physiological defences are not specifically regulated by the IIS pathway and DAF-16, but expression of DAF-16 target genes in IIS mutants and dauers is sufficient to promote cross tolerance to low temperatures in addition to other forms of stress.

## Introduction

Genetic modifications which promote longevity are associated with increased resistance to environmental stress in model organisms ranging from yeast to mice. These longevity and stress-resistant phenotypes can be induced by disrupting conserved signal transduction pathways which respond to changes in nutrient availability and/or other environmental conditions (reviewed in [1]). Such responses are mediated by a physiological shift towards protection and repair of somatic molecules and cells. The most extensively characterised of these pathways is the conserved insulin/IGF-1 signalling (IIS) pathway, which plays an important role in the determination of longevity and stress resistance in *Caenorhabditis elegans* [2], [3], [4], [5], *Drosophila melanogaster* [6], [7] and mice, *Mus musculus* [8], and may also influence lifespan in humans [9], [10].

In *C. elegans*, the IIS pathway modulates development, metabolism, stress resistance and longevity, at least in part, by regulating the activity of the FOXO transcription factor DAF-16. The IIS pathway negatively regulates DAF-16 activity in favourable growth conditions [11], [12]. However, when insulin/IGF-1 signalling is disrupted by low food availability or exposure to certain forms of stress, DAF-16 accumulates within nuclei, binding to and activating the promoters of genes involved in cellular defence and metabolism [11], [12], [13], [14]. Mutants which are defective for components of the IIS pathway such as DAF-2, the insulin/IGF-1 receptor homologue, or AGE-1, the phosphatidylinositol 3-kinase (PI3K) catalytic subunit homologue, are long-lived and display increased resistance to a range of environmental challenges, including oxidative stress [4], [15], heat shock [5], ultraviolet light [16], heavy metals [17], hypoxia [18], microbial infections [19], and hypertonic stress [20].

Although the increased activity of DAF-16 in IIS mutants appears to induce a physiological response promoting cross-tolerance to multiple forms of stress, some forms of stress have not yet been associated with the IIS pathway. Artificial selection for increased longevity promotes cold tolerance in *D. melanogaster* [21], [22], but resistance to low temperatures, which may be an important survival trait, has not been reported for any IIS mutant. In fact, in *D. melanogaster*, ablation of neurosecretory cells, which produce insulin-like ligands required for activation of the IIS pathway, increases lifespan, but delays cold shock recovery [23]. Although the ablated animals do display enhanced resistance to starvation and oxidative stress, reduced insulin/IGF-1 signalling may actually increase sensitivity to low temperatures in flies.

Whilst molecular chaperones (heat-shock proteins) are known to play a key role in preventing and repairing damage induced by high temperatures [24], the preservation of cell membrane fluidity is thought to be important for cold tolerance in poikilotherms. At physiological temperatures to which organisms are either adapted or acclimated, membrane lipids are maintained in a fluid or liquid-crystalline phase. However, when temperatures drop below a threshold level, lipid structure changes to a more ordered, rigid gel phase which impairs vital membrane functions [25]. To promote survival at low temperatures, poikilotherms can reduce the average temperature at which this transition occurs by increasing the proportion of unsaturated fatty acids in membrane phospholipids [26]. This response is at least partially mediated by the activity of Δ^9^ desaturase enzymes [27], [28]. Δ^9^ desaturase genes have been implicated in cold tolerance in bacteria [29], plants [30], and poikilothermic animals [31], [32], [33].

At favourable growth temperatures, expression of the three *C. elegans* Δ^9^ desaturase genes *fat-5*, *fat-6* and *fat-7* is regulated by the IIS pathway and DAF-16 [14], and *fat-6* has been identified as a direct target of DAF-16 [34]. All three genes are up-regulated in IIS mutants and during the facultative, *daf-16*-dependent, dauer stage [14], [35], [36], [37]. Consequently, membrane fluidity should be maintained to lower temperatures in non-dauer IIS mutants than in wild type due to a higher proportion of unsaturated fatty acids in membrane phospholipids. This may enhance survival of IIS mutants following a rapid temperature decline. In this study we compared survival of long-lived *age-1(hx546)* mutant and wild type *C. elegans* at low temperatures, and assessed the contribution of Δ^9^ desaturase genes to cold tolerance in each genotype.

## Results

### Reduced insulin/IGF-1 signalling promotes cold tolerance in a *daf-16* dependent manner

**Figure 1:**
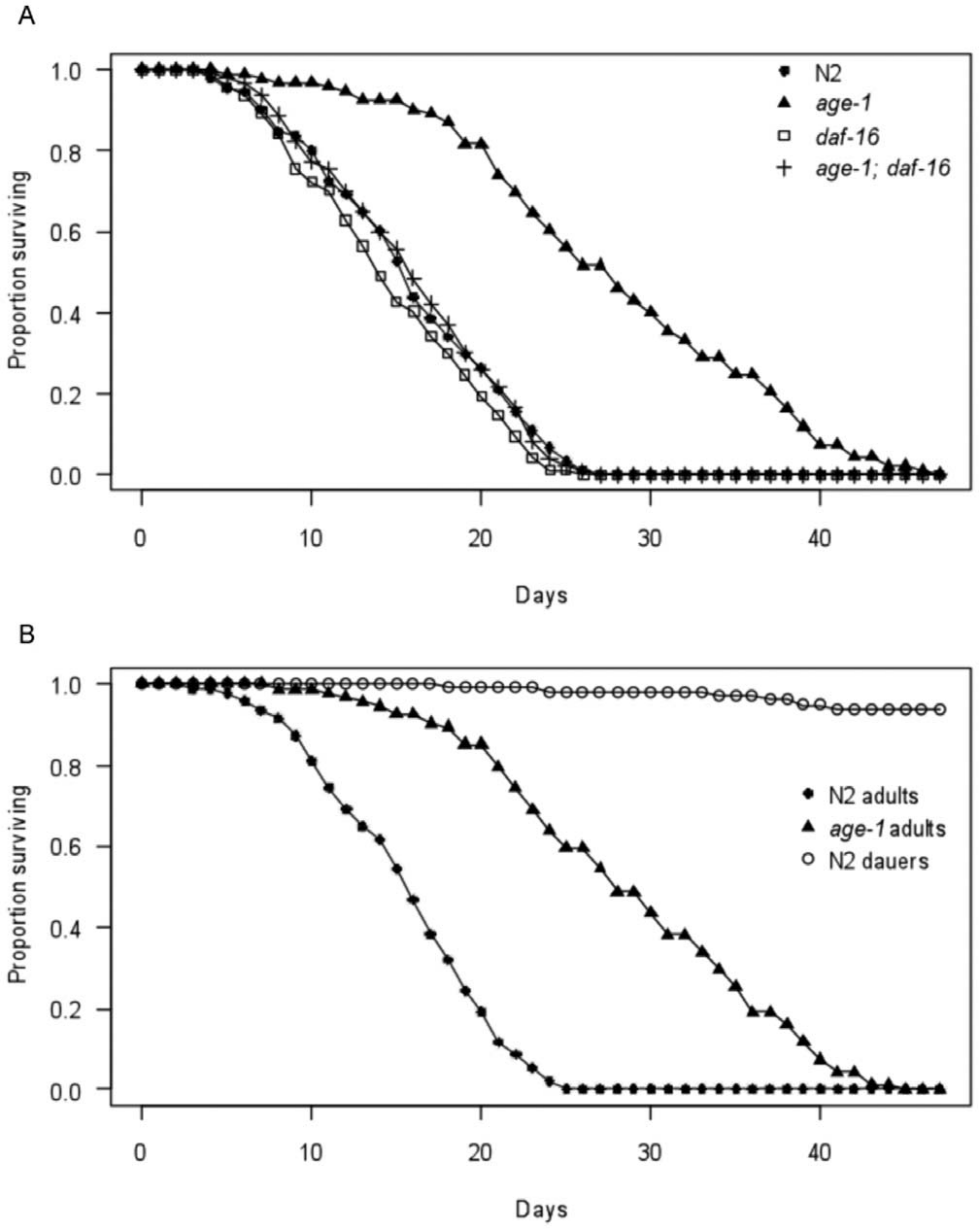
Survival curves following direct transfer from 20°C to 4°C. Survival curves at 4°C for A) wild type (N2), *age-1(hx546)* mutants, *daf-16(mu86)* mutants and *age-1(hx546)*; *daf-16(mu86)* double mutants, and B) wild type (N2) and *age-1(hx546)* mutant adults and wild type (N2) dauers (n = 90–100 per genotype and stage).

To establish if insulin/IGF-1 signalling plays a role in cold tolerance, survival times were compared between young wild type (N2) and *age-1(hx546)* mutant adults following direct transfer from 20°C to 4°C±0.5°C. This is substantially below the thermal range for wild type growth and activity of 15°C–25°C [38], and the lipid phase transition temperature of approximately 10°C [39]. Survival was considerably prolonged in *age-1(hx546)* mutants relative to wild type adults under these conditions (z = 12.02, p<0.001), with mean survival times increased by approximately 85% (Figure 1A). This indicates that these long-lived worms display increased resistance to low temperatures in addition to other forms of stress. To assess if the discrepancy in cold tolerance between the two genotypes was mediated by differences in the activity of DAF-16, survival times were also monitored in *daf-16(mu86)* null mutants and in *age-1(hx546)*; *daf-16(mu86)* double mutants following direct transfer from 20°C to 4°C±0.5°C. No significant differences in survival were observed among wild type, *daf-16(mu86)* mutants and *age-1(hx546)*; *daf-16(mu86)* double mutants under these conditions (Figure 1A). This suggests that *daf-16* is required for increased cold tolerance of *age-1(hx546)* mutants, but does not contribute to survival of wild type adults at low temperatures.

Dauer formation is partially regulated by the IIS pathway and is dependent upon *daf-16* [3]. As Δ^9^ desaturase genes are up-regulated in wild type dauers, we also examined resistance to low temperature stress during this stage. Over 90% of dauers survived at 4°C±0.5°C until after all fed wild type and *age-1(hx546)* mutant adults had died (Figure 1B). This indicates that dauers are remarkably resilient to cold temperatures, as to other stresses, relative to wild type adults (z = 13.49, p<0.001), and that cold tolerance in *age-1(hx546)* mutant adults is lower than in wild type dauers under these conditions (z = 8.85 p<0.001).

### Cold tolerance in *age-1(hx546)* mutants is facilitated by Δ^9^ desaturases

The *C. elegans* Δ^9^ desaturase enzymes exhibit slight differences in substrate specificity, with FAT-5 primarily converting palmitic acid to palmitoleic acid, whilst FAT-6 and FAT-7 primarily convert stearic acid to oleic acid [40]. Despite this, due to considerable overlap in biochemical activity, loss-of-function mutations in either *fat-6* or *fat-7* induce compensatory responses in the expression of the remaining Δ^9^ desaturase genes, and mutants that are defective for single Δ^9^ desaturase genes display no obvious phenotype [41]. Therefore, to assess the extent to which Δ^9^ desaturase enzymes contribute to cold tolerance in *age-1(hx546)* mutants, the expression of multiple Δ^9^ desaturase genes was simultaneously eliminated or suppressed using a combination of loss-of-function mutations and RNA interference (RNAi).

**Figure 2:**
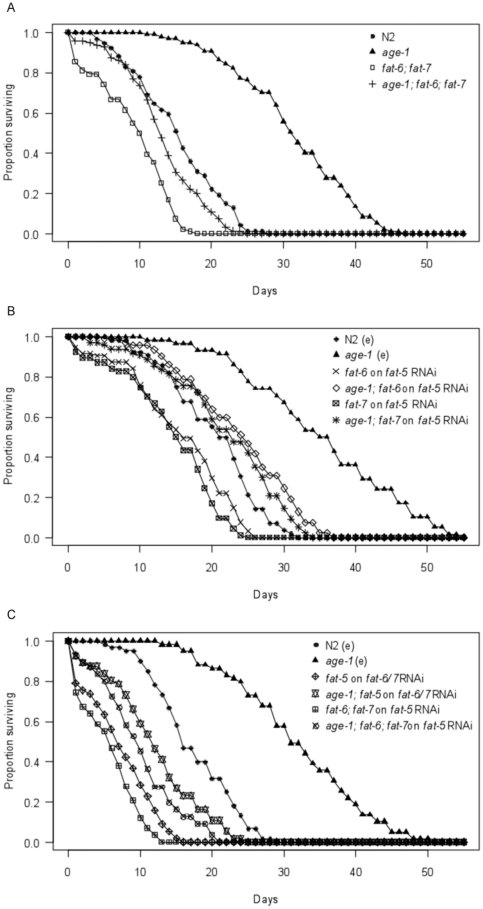
Contribution of Δ9 desaturase genes to survival following direct transfer from 20°C to 4°C. Survival curves at 4°C for A) wild type (N2), *age-1(hx546)* mutants, *fat-6(tm331)*; *fat-7(wa36)* double mutants and *age-1(hx546)*; *fat-6(tm331)*; *fat-7(wa36)* triple mutants (n = 90–100 per genotype), B) wild type (N2) and *age-1(hx546)* mutant controls on empty vector HT115 bacteria (N2(e) and *age-1*(e)), and *fat-6(tm331)* mutants, *fat-7(wa36)* mutants, *age-1(hx546)*; *fat-6(tm331)* double mutants and *age-1(hx546)*; *fat-7(wa36)* double mutants on *fat-5* RNAi bacteria (n = 50–60 per genotype on empty vector HT115 bacteria, n = 90–100 per genotype on *fat-5* RNAi bacteria), C) wild type (N2) and *age-1(hx546)* mutant controls on empty vector HT115 bacteria, *fat-6(tm331)*; *fat-7(wa36)* double mutants and *age-1(hx546)*; *fat-6(tm331)*; *fat-7(wa36)* triple mutants on *fat-5* RNAi bacteria, and *fat-5(tm420)* mutants and *age-1(hx546)*; *fat-5(tm420)* double mutants on *fat-6*/*fat-7* RNAi bacteria (n = 50–60 individuals per genotype/treatment).

The *fat-6(tm331)*; *fat-7(wa36)* double mutant had dramatically reduced survival times at 4°C in the wild type (z = −7.79, p<0.001) and *age-1(hx546)* mutant (z = −14.5, p<0.001) backgrounds relative to respective controls (Figure 2A). Whilst mean survival times were reduced by 40% in the wild-type background, mean survival was reduced by almost 58% in the *age-1(hx546)* mutant background, suggesting that *fat-6* and *fat-7* play an important role in the increased cold tolerance of these long-lived mutants. The *age-1(hx546)*; *fat-6(tm331)*; *fat-7(wa36)* triple mutants were moderately more sensitive to low temperatures than wild type worms (z = −2.53, p = 0.012), with mean survival times reduced by almost 14%. However, *fat-6(tm331)*; *fat-7(wa36)* individuals with an *age-1* mutant background were more cold tolerant than those with a wild type background (z = 5.27, p<0.001), with mean survival times remaining approximately 43% greater. This suggests that changes induced by the *age-1(hx546)* mutant allele which promote cold tolerance involve another gene or genes, in addition to *fat-6* and *fat-7*.

Relative to wild type controls, RNAi of *fat-5* in *fat-6(tm331)* mutants reduced mean survival times at 4°C (z = −3.48, p<0.001) by almost 24%, and RNAi of *fat-5* in *fat-7(wa36)* mutants reduced mean survival times at 4°C (z = −4.84, p<0.001) by 30% (Figure 2B). Similarly, RNAi of *fat-5* reduced mean survival times in *age-1(hx546)*; *fat-6(tm331)* double mutants (z = −6.67, p<0.001) by approximately 33%, and in *age-1(hx546)*; *fat-7(wa36)* double mutants (z = −7.81, p<0.001) by 38%, relative to *age-1(hx546)* mutant controls (Figure 2B). However, both single *fat-6(tm331)* or *fat-7(wa36)* desaturase mutants with an *age-1(hx546)* mutant background subjected to *fat-5* RNAi remained considerably more cold tolerant than equivalent individuals in a wild type background (*fat-6(tm331)* mutants: z = 7.12, p<0.001, *fat-7(wa36)* mutants: z = 7.49, p<0.001). In both cases mean survival times remained approximately 52% greater in the *age-1(hx546)* mutant background.

Simultaneous mutations in all three Δ^9^ desaturase genes cause embryonic lethality [41]. Therefore, to obtain individuals in which the expression of the three Δ^9^ desaturase genes had been reduced or eliminated, RNAi was used in two different strategies. First, *fat-5* expression was reduced in *fat-6(tm331)*; *fat-7(wa36)* double mutants and in *age-1(hx546)*; *fat-6(tm331)*; *fat-7(wa36)* triple mutants. Second, *fat-6* and *fat-7* expression was reduced in *fat-5(tm420)* mutants and in *age-1(hx546)*; *fat-5(tm420)* double mutants. As *fat-6* and *fat-7* have approximately 84% nucleotide homology, the expression of both genes is suppressed when either is targeted by RNAi [41].

When the function of all three Δ^9^ desaturase genes was reduced or eliminated, survival times at 4°C were reduced dramatically in both the wild type and *age-1(hx546)* mutant backgrounds relative to controls (Figure 2C). Compared to wild type controls, RNAi of *fat-5* in *fat-6(tm331)*; *fat-7(wa36)* double mutants reduced mean survival times (z = −10.60, p<0.001) by 67%, and RNAi of *fat-6* and *fat-7* in *fat-5(tm420)* mutants reduced mean survival times (z = −8.23, p<0.001) by approximately 58%. Relative to *age-1* mutant controls, RNAi of *fat-5* in *age-1(hx546)*; *fat-6(tm331)*; *fat-7(wa36)* triple mutants reduced mean survival times (z = −11.40, p<0.001) by almost 69%, and RNAi of *fat-6* and *fat-7* in *age-1(hx546)*; *fat-5(tm420)* double mutants reduced mean survival times (z = −9.58, p<0.001) by 62%. In both genetic backgrounds, *fat-6(tm331)*; *fat-7(wa36)* mutants subjected to *fat-5* RNAi appeared more sensitive to cold than *fat-5(tm420)* mutants subjected to *fat-6*/*fat-7* RNAi. However, these differences were not statistically significant and, even if real, may simply reflect reduced efficiency of RNAi relative to loss-of-function mutations. Importantly, individuals with an *age-1(hx546)* mutant background remained more cold tolerant than equivalent individuals with a wild type background even when the expression of all three Δ^9^ desaturase genes had been knocked out or knocked down. When *fat-5* expression was suppressed by RNAi in *age-1(hx546)*; *fat-6(tm331)*; *fat-7(wa36)* triple mutants, mean survival times were higher than in *fat-6(tm331)*; *fat-7(wa36)* double mutants (z = 5.35, p<0.001) by approximately 75%. Similarly, mean survival times remained greater in *age-1(hx546)*; *fat-5(tm420)* double mutants on *fat-6*/*fat-7* RNAi plates than in *fat-5(tm420)* mutants on *fat-6*/*fat-7* RNAi plates (z = 4.92, p<0.001) by 66%. These results again suggest that Δ^9^ desaturase genes play an important role in the cold tolerant phenotype of *age-1(hx546)* mutants, but indicate that additional genes are also involved.

### Δ^9^ desaturase enzymes promote rapid recovery from cold shock

**Figure 3:**
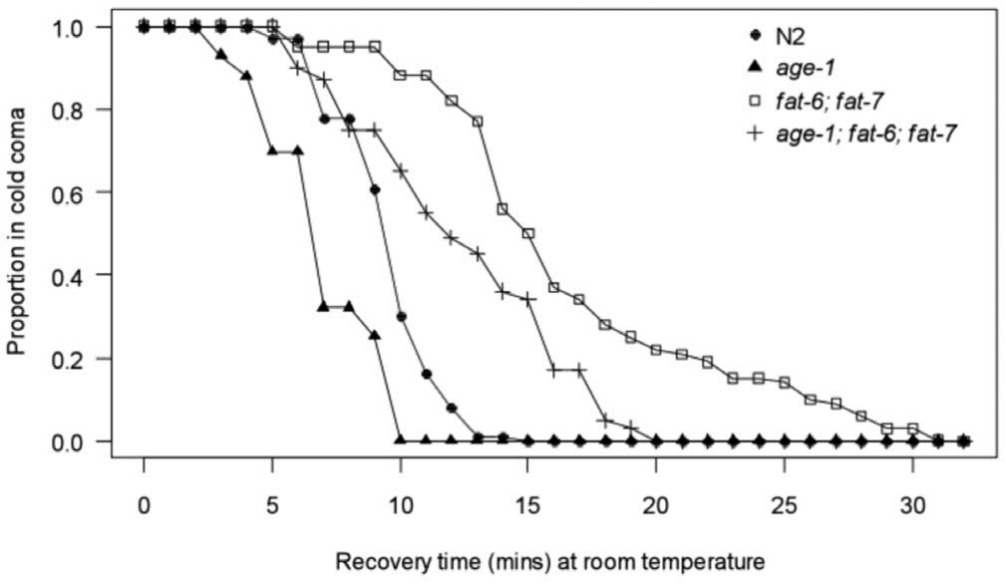
Cold coma recovery as a measure of cold tolerance. Variation in recovery times among wild type (N2), *age-1(hx546)* mutants, *fat-6(tm331)*; *fat-7(wa36)* double mutants and *age-1(hx546)*; *fat-6(tm331)*; *fat-7(wa36)* triple mutants following 6 hours cold shock at 4°C (n = 95–100 individuals per genotype, observed over 2 separate blocks).

‘Cold coma’ recovery times have been used to characterise variability in cold tolerance among different *D. melanogaster* strains [23], [42]. To determine if this approach can be used to assess differences in cold sensitivity among *C. elegans* genotypes, we compared recovery times at room temperature (∼22°C), following 6 hours exposure to 4°C±0.5°C, among a representative set of strains; wild type (N2), *age-1(hx546)* mutants, *fat-6(tm331)*; *fat-7(wa36)* double mutants and *age-1(hx546)*; *fat-6(tm331)*; *fat-7(wa36)* triple mutants. Considerable variation in cold coma recovery times was observed among the different genotypes (Figure 3). Although *age-1(hx546)* mutants resumed locomotion more rapidly than wild type worms after removal from the low temperature stress (t = 7.18, p<0.001), with mean recovery times reduced by almost 27%, differences in recovery times between the two genotypes were less apparent than differences in survival following prolonged periods at 4°C. However, the *fat-6(tm331)*; *fat-7(wa36)* double mutant had substantially delayed recovery times in the wild type (t = −12.37, p<0.001) and *age-1(hx546)* mutant (t = −12.65, p<0.001) backgrounds relative to respective controls. Mean recovery times were prolonged by 74% in the wild type background and by almost 76% in the *age-1(hx546)* mutant background. FAT-6 and/or FAT-7 thus promote recovery of locomotion in both genotypes following exposure to low temperatures. Although recovery was delayed in *age-1(hx546)*; *fat-6(tm331)*; *fat-7(wa36)* triple mutants relative to the wild type strain (t = −6.07, p<0.001), these worms recovered more rapidly than *fat-6(tm331)*; *fat-7(wa36)* double mutants with a background that was wild type for *age-1* (t = 6.89, p<0.001), with mean recovery times reduced by approximately 26%. These results reinforce the findings from the survival experiments (Figure 2A) concerning the contribution of *fat-6*, *fat-7* and other genes to tolerance of cold stress in *age-1(hx546)* mutants, and exemplify the use of cold coma recovery assays to assess variation in cold tolerance among *C. elegans* genotypes.

### DAF-16 is not activated during exposure to low temperatures

**Figure 4:**
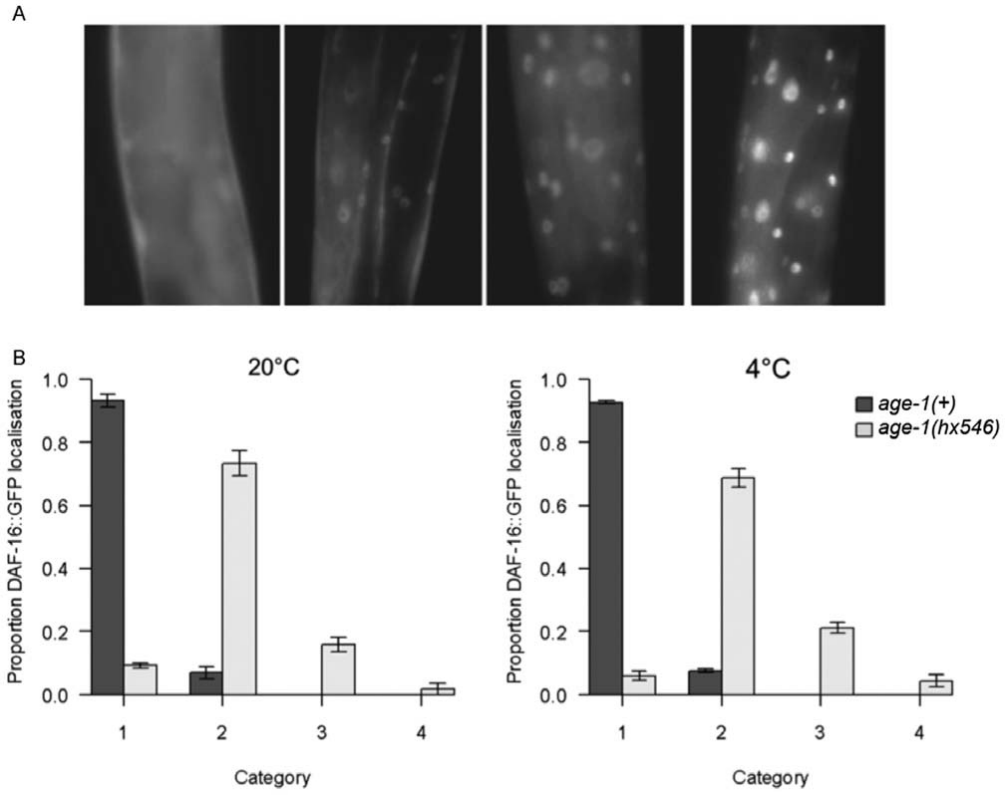
Subcellular localization of DAF-16::GFP in response to cold stress. A) Subcellular distributions of DAF-16::GFP were categorised with a score from 1 (left) with no nuclear localization to 4 (right) with complete nuclear localization. B) The mean proportion of *age-1(+)* adults and *age-1(hx546)* mutant adults which displayed categories 1–4 of DAF-16::GFP localisation at 20°C (left) and 4°C (right). Error bars represent standard errors of the means (n = 100–120 per genotype and treatment observed over 3 separate blocks).

As functional *daf-16* does not appear to be required for cold tolerance in wild type adults, we asked specifically if the DAF-16 transcription factor becomes activated at low temperatures. Cellular distributions of a DAF-16::GFP fusion protein were categorised on a scale ranging from 1 (unlocalised), representing minimal DAF-16 activity, to 4 (fully nuclear localised), representing maximal DAF-16 activity (Figure 4A). Distributions were scored in young adults following 6 hours exposure to 4°C, and were compared with controls maintained at 20°C. The 6 hour stress period was considered to be a sufficient duration as a 20-fold increase in *fat-7* expression has been reported in wild type *C. elegans* within 3 hours of transfer from 25°C to 10°C [33]. Whilst the cellular distribution of DAF-16::GFP was predominantly unlocalised in *age-1(+)* adults at 20°C, accumulation of DAF-16::GFP was observed in the nuclei of *age-1(hx546)* mutants (category 1–2: t = 17.26; no *age-1(+)* individuals displayed categories 3 or 4, p<0.001) (Figure 4B). Although there was a slight increase in the intensity of nuclear localisation in *age-1(hx546)* mutant adults at 4°C (category 2–3: t = 2.79, p = 0.029), no significant change in the subcellular localisation of DAF-16::GFP was observed in *age-1(+) C. elegans* at this temperature relative to controls maintained at 20°C (Figure 4B). These observations indicate that wild type *C. elegans* does not respond to cold conditions specifically through the IIS pathway and DAF-16. It is as yet unknown how the genes involved in delivering cold tolerance are regulated in response to acclimation to cold conditions in non-dauer wild type *C. elegans*. Nevertheless, some of the targets of the IIS pathway and DAF-16 which are up/down regulated in *age-1(hx546)* mutants clearly overlap with the mechanism(s) required for cold resistance.

## Discussion

It is well established that mutations which disrupt insulin/IGF-1 signalling promote longevity and stress resistance in diverse evolutionary lineages. However, no assessment of cold tolerance has previously been reported in any IIS mutant of any species, despite this presumably being an important component of life history in many wild populations. We have demonstrated that *age-1(hx546)* mutants have increased cold tolerance relative to wild type *C. elegans* when transferred directly from an optimal growth temperature to 4°C during early adulthood. As activity and reproduction completely arrest at this temperature, survival at 4°C does not simply reflect longevity under optimal conditions. We have also established for the first time that dauer larvae can survive for prolonged periods at low temperatures compared to adults which have undergone development in favourable growth conditions, which may represent a survival strategy in seasonal temperate environments.

### Δ^9^ desaturase genes promote cold tolerance in *age-1(hx546)* mutants

The cold tolerant phenotype of *age-1(hx546)* mutants and dauers may arise because these worms are physiologically prepared for the stress, at least with regard to the threat to membrane function, through changes in membrane lipid composition brought about by the up-regulation of Δ9 desaturase genes. Both a cold tolerant phenotype reduction and a prolongation of cold coma recovery time arise when Δ9 desaturase expression is experimentally disrupted in *age-1(hx546)* mutants. Tolerance of 4°C appeared reduced to a greater extent when both the *fat-6* and *fat-7* Δ9 desaturase genes were defective as compared to when *fat-5* and *fat-6* or *fat-5* and *fat-7* expression was eliminated or suppressed. Consistent with this, Brock *et al.* [31] demonstrated the same relationship for sensitivity to 10°C and 15°C. The *fat-6(tm331)*; *fat-7(wa36)* double mutants also displayed additional defects, including reduced fat storage, slow growth, reduced fecundity and a high proportion of embryonic lethality, despite a more than 40-fold increase in *fat-5* expression [31]. Presumably these observations reflect the close evolutionary relationship and substrate specificity of the FAT-6 and FAT-7 Δ9 desaturases, and the distinct specificity of the FAT-5 Δ9 desaturase [40]. When the expression of all three Δ9 desaturase genes was eliminated or suppressed, low temperature resistance was reduced more dramatically in the *age-1(hx546)* mutant than the wild type background, but the *age-1* mutation still conferred some elevation of cold tolerance. This suggests that while the cold tolerance of *age-1(hx546)* mutants is principally due to the Δ9 desaturase genes, additional genes contribute to the phenotype. Mutations in *age-1* may cause changes in the expression of other genes encoding enzymes involved in lipid metabolism that may also affect membrane fluidity [43].

### Additional DAF-16 target genes may contribute to cold tolerance

IIS mutants and dauers differ from wild type adults in other aspects of metabolism which may be involved in cold tolerance. For instance, genes involved in the synthesis of trehalose are up-regulated in *daf-2(e1370)* mutants and dauers [36], [37], [44], and trehalose levels are approximately 2-fold higher in *age-1(hx546)* mutants than in the wild type [20]. Trehalose sugars have been implicated in cold acclimation and/or cold tolerance in yeast [45], nematodes [46] and insects [47], [48], [49]. Glycerol, which has a well established role as a cryoprotectant and has been implicated in rapid cold-hardening in several insect species [50], is also found at higher levels in *age-1(hx546)* mutants than in wild type *C. elegans* [20]. Trehalose and glycerol may also be involved in modifying the membrane lipid phase transition and directly affecting membrane fluidity [51], [52], and so could act in combination with Δ^9^ desaturase enzymes to enhance the preservation of membrane function at low temperatures in *age-1(hx546)* mutants.

Murray *et al.* [33] demonstrated that acquired cold tolerance in *C. elegans* following a period of acclimation at 10°C is not exclusively dependent upon membrane lipid composition, and that additional mechanisms must contribute to cold acclimation and cold tolerance in poikilothermic animals. Heat-shock proteins enhance cold tolerance in *S. cerevisiae* [53] and in a variety of insect species [54], and antioxidant enzymes have been implicated in resistance to low temperatures in insects [55] and plants [56], [57]. Several genes encoding heat-shock proteins are expressed at higher levels in IIS mutants and dauers than in wild type adults [14], [35], [37], and certain antioxidants are present at higher levels in *age-1(hx546)* mutants and dauers than in wild type adults [4]. These may make a contribution to, or fully explain, the additional cold tolerance phenotype beyond that provided by the desaturase genes in the *age-1(hx546)* mutants.

### DAF-16 mediates a general rather than a specific stress response

In contrast to high temperature and oxidative stress [58], *daf-16(mu86)* mutants are as sensitive to low temperatures as wild type adults. Additionally, no activation of DAF-16, as revealed by nuclear localisation of DAF-16::GFP, was observed when individuals with a wild type background were exposed to low temperatures. This suggests that insulin/IGF-1 signalling and DAF-16 activation are not required for the expression of genes in physiological defence against cold-induced damage. RNAi of *fat-6* and *fat-7* also increase resistance to high temperatures in wild type *C. elegans* independently of *daf-16* [59], another example of a stress defense mechanism mediated by lipid metabolism with and without DAF-16 activity.

Using a line transgenic for a *daf-16::gfp* fusion gene [12] different to the one used in this study [11], Wolf *et al.* [60] reported moderate activation of DAF-16, as revealed by increased nuclear localization, following 2 hours exposure to 1°C or 8°C. However, they also observed nuclear localisation of DAF-16::GFP in approximately 40% of adults with a wild type background at the optimal growth temperature of 20°C. Cold exposure has been shown to modify insulin/IGF-1 signalling in a tissue specific manner in rats, with signal transduction being reduced in the hypothalamus, skeletal muscle and white adipose tissue, but enhanced in brown adipose tissue [61], [62]. Though it remains unclear which transgenic *C. elegans* strain most accurately portrays changes in subcellular distribution of DAF-16 under different thermal conditions, cold tolerance in wild type *C. elegans* has also been shown here to be *daf-16* independent.

DAF-16::GFP translocates to the nucleus in response to high temperatures, oxidative stress and starvation [11], but remains unlocalized upon exposure to ultraviolet light [11], hypertonic stress [20], and *Pseudomonas aeruginosa* infection [63]. Like the findings presented here, long-lived IIS mutants display increased resistance to ultraviolet light, hypertonic stress and pathogen infections which is dependent upon *daf-16*, yet functional *daf-16* is not required for wild type levels of resistance to these conditions [16], [19], [20]. Although different forms of stress may require distinct responses, mediated by different signalling pathways and transcription factors, an overlap may be expected in the stress response proteins involved [64]. Consequently, *daf-16* independent mechanisms which target effector genes with expression up or down regulated in IIS mutants and dauers may be involved in mediating the cold-induced stress response. Several transcription factors, in addition to DAF-16, are known to regulate Δ^9^ desaturase gene expression, including the nuclear hormone receptors NHR-49 and NHR-80, and SBP-1, the *C. elegans* homologue of sterol-regulatory-element-binding-protein (SREBP) transcription factors, key regulators of lipid homeostasis in mammals [41], [65], [66], [67]. It is conceivable that one or more of these may play an important role in cold tolerance and/or cold acclimation in *C. elegans*. One or more of these transcription factors or yet another transcription factor may well contribute to cold tolerance in *age-1(hx546)* mutants, including through the activation of the desaturase genes. However, as the increased cold tolerant phenotype of *age-1(hx546)* mutants is entirely dependent upon functional *daf-16*, all the additional resistance to cold stress exhibited by the *age-1(hx546)* mutants appears to be mediated by DAF-16 activity.

### Evolutionary conservation of longevity and stress resistance mechanisms

We have established for the first time that an association between longevity and resistance to low temperatures exists in *C. elegans*. Such a relationship has previously been observed in *D. melanogaster* lines selected for increased longevity [21], [22]. Although we found that reduced insulin/IGF-1 signalling in *age-1(hx546)* mutants promotes cold tolerance, Broughton *et al.* [23] reported that increased longevity induced by the ablation of neurosecretory cells which produce insulin-like peptides is associated with cold sensitivity in *D. melanogaster*. However, the ablated flies displayed reduced levels of the cryoprotectant trehalose in the hemolymph, while trehalose levels are up-regulated in *C. elegans* IIS mutants and dauer larvae [20], [36], [37], [44]. This discrepancy presumably reflects differences in the downstream targets of the IIS pathway in different species.

The higher cold tolerance of dauers may be important for survival in *C. elegans* populations that experience rapid fluctuations in temperature. In some invertebrates diapause is the primary mechanism for over-winter survival, and is induced in response to changes in temperature and/or photoperiod (reviewed in [68], [69]). In *D. melanogaster* and the mosquito *Culex pipiens*, this appears to be regulated by the IIS pathway and orthologues of DAF-16 [7], [70], [71]. However, in contrast to high temperatures [72], there is no evidence to suggest that dauer formation in *C. elegans* can be induced by exposure to cold conditions. Dauers presumably exhibit cold tolerance due to the overlap in the effector genes targeted by both the IIS pathway/DAF-16 and, by the cold induced stress response. Overlaps in effector genes for different stress conditions may facilitate evolutionary change in stress responses, allowing adaptation to changes in the spectrum of stress conditions encountered.

Associations between lifespan and stress resistance in mutants with reduced insulin/IGF-1 signalling have been observed in a variety of model organisms. As insights into the mechanisms which underlie such associations contribute to our understanding of longevity and stress resistance, it will be interesting to establish if the relationship between lifespan and cold tolerance identified in *C. elegans* IIS mutants has been conserved.

## Materials and Methods

### Strains and culture conditions

The following genotypes were obtained from the Caenorhabditis Genetics Centre: N2 Bristol (wild type), TJ1052 *age-1(hx546)*, CF1038 *daf-16(mu86)*, BX107 *fat-5(tm420)*, BX106 *fat-6(tm331)*, BX153 *fat-7(wa36)*, BX160 *fat-6(tm331)*; *fat-5(tm420)*, BX110 *fat-7(wa36)*; *fat-5(tm420)*, BX156 *fat-6(tm331)*; *fat-7(wa36)* and TJ356 N2; *zIs356 (daf-16p::daf-16a::gfp; rol-6[su1006])*. Long-lived *age-1(hx546)* mutants were utilized due to their ability to develop and reproduce at temperatures which induce developmental arrest in other IIS mutants. Crosses between *age-1(hx546)* mutants and additional strains were confirmed by PCR using gene specific primers listed in Table S1. Except where stated otherwise, strains were maintained on nematode growth media (NGM) plates, containing 10 µg/ml nystatin and 50 µg/ml streptomycin, and seeded with *E. coli* strain HB101. Strains were maintained at 20°C until cold tolerance assays commenced.

### RNAi

RNA interference (RNAi) was induced by feeding using clones from the Ahringer library. RNAi experiments were performed on NGM plates supplemented with Ampicillin, Tetracycline and IPTG as previously described [73]. *C. elegans* fed on HT115 bacteria containing an empty RNAi plasmid vector (pL4440) were used as negative controls.

### Cold tolerance assays

Age-synchronised adults, on the first day of reproduction, were transferred directly from 20°C to 4°C±0.5°C on plates containing approximately 20 individuals each. Survival was monitored regularly, following 20–30 minutes recovery at room temperature, until all individuals had died. If survival status was not immediately apparent, worms were gently touched with a platinum wire to stimulate a response.

Dauer larvae were transferred directly from 20°C to 4°C±0.5°C approximately 24 hours after populations had become starved. Survival status was monitored in 100 individuals, divided among 5 NGM plates, until all non-dauer controls had died.

### Cold coma recovery times

Age-synchronised adults, on the first day of reproduction, were transferred from 20°C to 4°C±0.5°C for 6 hours on NGM plates containing 10 individuals each. After the stress period, recovery times were monitored at room temperature (approximately 22°C). Individuals were considered to have recovered when they began to move spontaneously around the NGM plates. Recovery times were recorded in 100 individuals per genotype over 2 separate blocks.

### DAF-16::GFP localisation

Cellular distributions of a DAF-16::GFP fusion protein were compared among well fed young adults which had been maintained at the control temperature (20°C) or exposed to 4°C±0.5°C for 6 hours. DAF-16::GFP localisation was categorised from 1–4, where 1 represents an unlocalised distribution throughout the cells and 2, 3 and 4 represent increasingly nuclear distributions, in 100–120 individuals per genotype and temperature treatment over 3 separate blocks. Worms were anaesthetized with 5 mM levamisole and epifluorescence imaging was performed using a Leica DMR HC confocal microscope.

### Statistical analyses

All analyses were performed in R version 2.10.1 [74].

For survival analysis, a small number of individuals which had died from causes other than exposure to low temperatures (e.g. rupturing of the vulva) were removed from the analysis. Each survival experiment was analysed independently using an accelerated failure time parametric regression model with a Weibull error distribution. These models were selected based upon visual inspection of the plotted residuals following analyses using a variety of different probability distributions. They were considered to be more robust than non-parametric survival models because the latter typically have greatest power when the hazard ratios (relative risks of death) between two covariates are proportional, and this was not the case with our data. However, to assess the generality of the results obtained from the accelerated failure time models, we also analysed the survival experiments using non-parametric weighted cox regression models, which provide unbiased average hazard ratio estimates rather than assuming proportional hazards. We obtained qualitatively very similar results from the two sets of analyses, but provide only the z-scores derived from the accelerated failure time models in the results section.

Cold coma recovery times were compared among genotypes using a generalised linear mixed effects model with a gamma error distribution. The model was fitted using the penalised quasi-likelihood (PQL) method [75], and included random effects terms to account for variation among NGM plates and between blocks.

DAF-16::GFP intracellular localisation scores were compared between genotypes and among temperature treatments using an ordinal multinomial continuation-ratio logit model [76]. When certain categories were not displayed by one or both genotypes following a particular thermal treatment, it was not possible to obtain parameters to describe all of the observed category transitions. These situations are described within parenthesis in the results. P-values were obtained using likelihood ratio tests to compare between models following sequential removal of explanatory variables and interactions.

## Supporting Information

Table S1 **Sequences of oligonucleotide primer pairs used in PCRs on genomic DNA to confirm** ***age-1*** **,** ***fat-5*** **,** ***fat-6*** **,** ***fat-7*** **and** ***daf-16*** **genotypes.**
(DOCX)
