# Supplementary material for: DAF-16 and Δ9 Desaturase Genes Promote Cold Tolerance in Long-Lived Caenorhabditis elegans age-1 Mutants
Source: PLoS One. 2011 Sep 8;6(9):e24550. doi: 10.1371/journal.pone.0024550 (PMC3169625; doi:10.1371/journal.pone.0024550)
Supplement: Table S1 — Sequences of oligonucleotide primer pairs used in PCRs on genomic DNA to confirm age-1 , fat-5 , fat-6 , fat-7 and daf-16 genotypes. (DOCX) [file pone.0024550.s001.docx]

Supplementary Table. Sequences of oligonucleotide primers used in this study.

| Gene (allele) | Nature of primer | Sequence (5’-3’) |
| --- | --- | --- |
|  |  |  |
| *age-1(hx546)* | upstream | CCAGTATTATGCCTGCTTCA |
|  | downstream | TGCGTACGGGTTCAAACAGC |
| *fat-5(tm420)* | upstream | AGACTCCGCCCCTTCTTTT |
|  | downstream (external) | AAGTGCTTTAGGCTTGGGCTC |
|  | downstream (internal) | CTGAATTAGGAAACGTAGGC |
| *fat-6(tm331)* | upstream | CCAGAGACGCAATATCTCGC |
|  | downstream (external) | CACATCCATGATTGGATACC |
|  | downstream (internal) | GATGAGCTCCGGCGGTTATT |
| *fat-7(wa36)* | upstream | ACAAGGCAACCACACCAATG |
|  | downstream | ATGCACCAAGTGGCGTGAAGT |
| *daf-16(mu86)* | upstream (internal) | CATCCATCCATACACCCACA |
|  | downstream (internal) | CAATTGATCGGTTGGCTTCT |
